# From hemocuprein to CSRP: the many faces of Cu/Zn superoxide dismutase

**DOI:** 10.1093/mtomcs/mfag007

**Published:** 2026-02-18

**Authors:** Ryan L Peterson, Valeria C Culotta

**Affiliations:** The Department of Chemistry and Biochemistry, Texas State University, San Marcos, TX 78666, United States; The Department of Biochemistry and Molecular Biology, Johns Hopkins University Bloomberg School of Public Health, Baltimore, MD 21205, United States

## Abstract

From bacteria to humans, the highly conserved Cu- and Zn-containing superoxide dismutase (Cu/Zn SOD) plays a pivotal role in free radical biology. By using Cu to disproportionate superoxide at rates that approach diffusion limits, Cu/Zn SODs are premier antioxidants. Interestingly, during eukaryotic evolution, several derivatives of the Cu/Zn SOD polypeptide appeared, where the Cu and/or Zn sites were lost and in some cases, Cu/Zn SOD-like sequences were replicated or fused to other protein domains. Such variations of Cu/Zn SOD include the CCS Cu chaperone, fungal Cu-only SODs, and animal CSRP (Cu-only SOD repeat proteins). Here we review the unique biophysical properties and biological functions of these Cu/Zn SOD-like proteins. CCS appeared early in eukaryotic evolution, where a primordial Cu/Zn SOD lost its Cu site and was fused to other Cu-binding domains, creating a dual Cu/molecular chaperone for intracellular Cu/Zn SOD. In the Opisthokont supergroup of eukaryotes that formed fungi and animals, a Cu/Zn SOD lost its Zn binding capacity and structural loop VII, forming Cu-only SODs of fungi and tandemly amplified Cu-only SODs in animal CSRP. Cu-only SODs and Cu-binding CSRPs are efficient SODs, and with lowered Cu-binding affinities, they have evolved to function exclusively outside the cell. Cu-only SODs promote virulence of pathogenic fungi, and recent studies have implicated a role for amphibian CSRP in tissue regeneration, a process involving reactive oxygen species. We have just begun to understand how nature has diversified the Cu/Zn SOD template to create new molecules for metal and free radical biology.

## Graphical Abstract

**Graphical Abstract:**
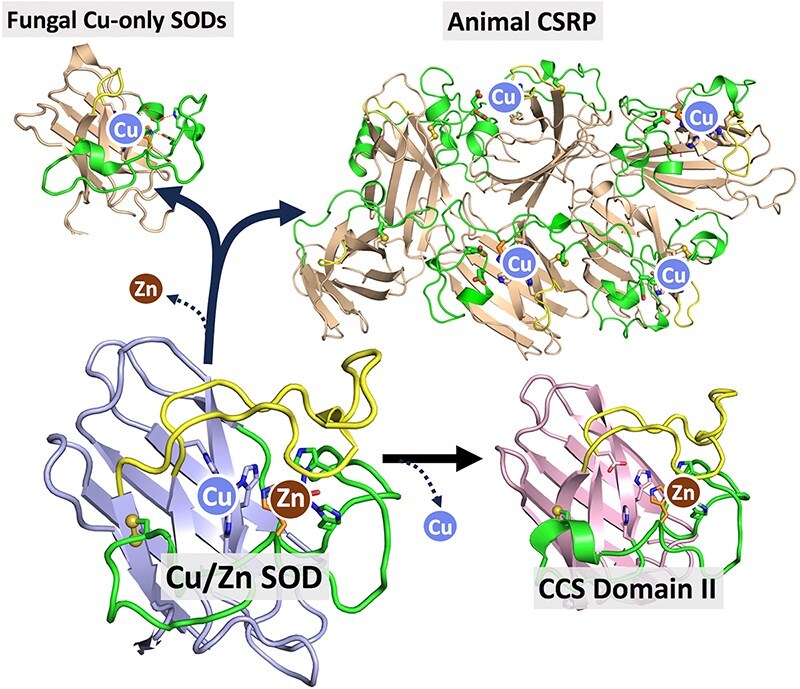
Model showing derivatives of Cu/Zn SOD in eukaryotes including the CCS copper chaperone, fungal Cu-only SODs and animal CSRP (Cu-only SOD repeat proteins)

## Introduction—the Cu/Zn SOD prototype

In 1969, Duke University Professor Irwin Fridovich and his PhD student at the time, Joe McCord, discovered a novel enzymatic activity for an abundant blue copper protein from bovine erythrocytes known as hemocuprein [1]. McCord demonstrated hemocuprein was capable of dismutating or disproportionating two molecules of superoxide anion to oxygen and H_2_O_2_ and therefore renamed the protein superoxide dismutase (SOD) [1].

This landmark discovery marked the birth of the field of reactive oxygen species (ROS) in biology.

The SOD identified by McCord and Fridovich disproportionates superoxide via two-step redox cycling of a catalytic Cu ion (see below).


\begin{eqnarray*}
{\mathrm{Cu}}\left( {{\mathrm{II}}} \right) + {{\mathrm{O}}_2}^{ \bullet -} \rightarrow {\mathrm{Cu}}\left( {\mathrm{I}} \right) + {{\mathrm{O}}_2}
\end{eqnarray*}

\begin{eqnarray*}
{\mathrm{Cu}}\left( {\mathrm{I}} \right) + {{\mathrm{O}}_2}^{ \bullet -} + 2{{\mathrm{H}}^ + } \rightarrow {\mathrm{Cu}}\left( {{\mathrm{II}}} \right) + {{\mathrm{H}}_2}{{\mathrm{O}}_2}
\end{eqnarray*}

\begin{eqnarray*}
{\mathrm{Net \,reaction}}:2{{\mathrm{O}}_2}^{ \bullet -} + 2{{\mathrm{H}}^ + } \rightarrow {{\mathrm{O}}_2} + {{\mathrm{H}}_2}{{\mathrm{O}}_2}
\end{eqnarray*}

Since then, SODs that use redox active Mn, Fe, or Ni ions have been identified [2–4]. Interestingly, the Mn/Fe and Ni binding SOD families represent completely distinct polypeptides bearing no commonality to the Cu containing SOD other than shared abilities to disproportionate superoxide at rates that approach diffusion limits [2, 5, 6]. With such incredible rates of reactivity, SODs are among those rare enzymes in biology labeled as “kinetically perfect”.

**Figure 1:**
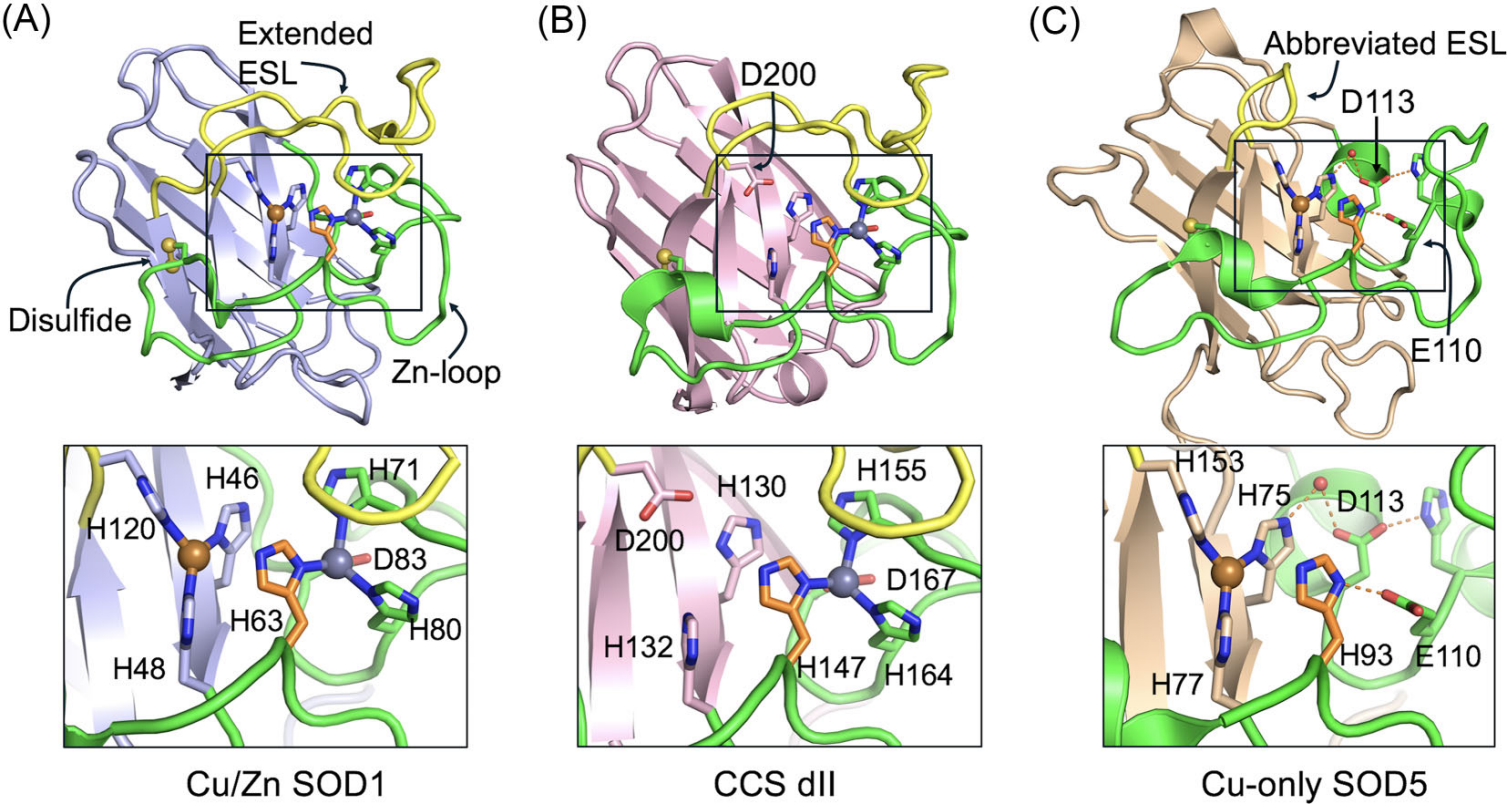
A structural comparison of Cu/Zn SOD versus CCS domain II versus Cu-only SODs. Comparison of the overall structures (top) and metal binding sites (bottom insets) of Cu/Zn SOD, CCS dII, and Cu-only SOD enzymes as determined by PyMol (version 3.1.6.1). The yellow, copper, and grey spheres correspond to the intramolecular disulfide bonds, Cu atoms, and Zn atoms, respectively. The copper binding histidines (SOD1 H46, H48, H63, and H120, and SOD5 H75, H77, H93, and H153), and zinc binding ligands (SOD1 H63, H71, H80, and D83, and CCS dII H147, H155, H164, and D167) are indicated, and the histidine equivalent that bridges Cu and Zn in SOD1 is colored orange. The Zn-loop is green, ESL is yellow, and the beta-barrel fold is colored purple (part A), pink (part B), or brown (part C). (A) The 3D structure of reduced *Saccharomyces cerevisiae* Cu/Zn SOD (PDB:2JCW). Note the extended ESL covering the active site. (B) The 3D structure of human CCS (PDB:6FN8). Note the D200 substitution for a Cu-binding histidine. (C) The 3D structure of reduced *Candida albicans* SOD5 (PDB:4N3T) highlighting the abbreviated ESL and open active site, and E110/D113 hallmark of active Cu-only SODs (see main text for details).

McCord and Fridovich’s Cu-SOD from bovine erythrocytes is actually a bimetallic enzyme containing both Cu and Zn with the Zn ion connected to the catalytic Cu via a bridging histidine ligand (Fig. 1A). While not directly participating in catalysis, Zn stabilizes the active site and the overall protein fold, and also helps maintain catalytic activity across wide ranges of pH [7, 8]. Other hallmarks of Cu/Zn SODs include an eight-stranded beta barrel backbone, a Zn-loop or loop IV that houses all of the Zn binding residues, an intramolecular disulfide, and an electrostatic loop (ESL) or loop VII structure covering the active site [9] (Fig. 1A). The positively charged ESL is believed to navigate superoxide free radicals towards the catalytic Cu and account for the remarkable rates of catalysis [10, 11].

Since its original identification in bovine serum, Cu/Zn SODs have been found throughout nature from bacteria to every cell in the human body. In eukaryotes, the intracellular so-called SOD1 version of the enzyme is largely cytosolic, but can also be found in the mitochondria, nucleus, and other organelles, and a separate extracellular Cu/Zn SOD can be found at the cell surface or is secreted to deal with extracellular sources of superoxide [12–15]. In bacterial species, Cu/Zn SODs are generally restricted to extracellular/periplasmic locations due to poor Cu availability inside the cell [16, 17].

Cu/Zn SODs are widely known as key cellular antioxidants [6, 18], but also play diverse roles in cell biology ranging from cell signaling involving ROS to transcriptional regulation in the nucleus [19, 20]. In humans, Cu/Zn SODs have been broadly implicated in health including impacts on aging, cancer, and cardiovascular disease [21–23], and mutations that affect Cu/Zn SOD1 protein stability have been linked to inherited disorders of amyotrophic lateral sclerosis [24, 25]. There are many excellent reviews that have been written on the topic of Cu/Zn SOD enzymes in cell biology and in health and disease [20, 21, 23, 26–28].

With the advent of genome sequencing in the late 1990s, Cu/Zn SOD sequences have appeared across the tree of life, but on occasion, some curious deviations from the bimetallic SOD prototype have been noted. The encoded proteins are not always predicted to bind Cu and/or Zn and certain key structural features of the enzyme may be missing. In other cases, the Cu/Zn SOD template is fused to other protein domains or present as repeated structures in large polypeptides. In this review, we shall focus on such variations of the Cu/Zn SOD polypeptide that have occurred in eukaryotic cells, specifically how nature has taken the bimetallic SOD blueprint and created distinct classes of eukaryotic molecules that function more broadly in metal metabolism, ROS biology, and cell fitness. Three eukaryotic protein families will be discussed including the CCS Cu chaperones, fungal Cu-only SODs, and the recently uncovered CSRP (Cu-only SOD repeat proteins) family of polypeptides in animals.

## CCS: Cu chaperone or molecular chaperone?

Due to the inherit toxicity of Cu in biological systems, bioavailable Cu inside cells is believed to be of extraordinarily low levels [29], posing a challenge to intracellular cuproproteins that rely on Cu for activity. To address this challenge, eukaryotes have evolved with “copper chaperones” to help deliver Cu to target enzymes [30–32]. Three major Cu chaperone classes were identified in the late 90s through genetic studies in the yeast *Saccharomyces cerevisiae*, namely ATX1 (human ATOX1) that delivers Cu to the Golgi for activation of secreted cuproenzymes [33]; COX17 for Cu delivery to the mitochondria for cytochrome c oxidase [34], and “CCS”, the Cu chaperone for Cu/Zn SOD1 [35].

CCS has three protein domains: a Cu-binding N-terminal domain resembling the ATX1 Cu chaperone, a small C-terminal oxidoreductase domain, and a larger internal domain II (“dII”) that structurally resembles SOD1 [36]. SOD1 is normally a homodimer and formation of a SOD1-CCS heterodimer through CCS dII allows direct transfer of Cu from CCS to SOD1 [37–39].

The resemblance of CCS dII to SOD1 is remarkably strong in the case of human CCS. The polypeptides share ≈50% amino acid identity [40], and CCS dII exhibits virtually all the hallmarks of a Cu/Zn SOD including the eight-stranded beta barrel fold and loop VII ESL, intramolecular disulfide and intact Zn binding site [41] (Fig. 1A, B). Three out of four Cu-binding histidines are present, while the fourth is replaced by an aspartate at CCS position 200. Without the fourth histidine, CCS dII is incapable of binding Cu and disproportionating superoxide [41–43]. In fact, a single D200H mutation converts human CCS into a Cu binding SOD enzyme that can self-activate itself with Cu [43]. It is worth noting that not all CCS dII molecules bear this striking resemblance to Cu/Zn SOD. *Saccharomyces cerevisiae* CCS dII is not readily recognizable as “SOD1-like” at the primary sequence level, but three-dimensional analysis clearly reveals hallmarks of the Cu/Zn SOD polypeptide including the eight-stranded beta-barrel fold [37, 39, 44, 45].

Since human CCS D200H can act as both a Cu chaperone and SOD enzyme, why has nature designed two separate molecules for Cu capture and SOD enzyme activity? As one possibility, dual molecules allow for extra layers of SOD control. Mammalian CCS is controlled by Cu at the level of protein turnover [46], and cells can therefore regulate SOD1 activity through CCS, without affecting SOD1 protein levels. As another possibility, CCS may have a function outside of Cu transfer. Indeed, CCS dII has been proposed to act as a molecular chaperone and help fold SOD1 into a conformer that favors Zn binding and disulfide oxidation [38, 39, 45, 47, 48]. This importance of CCS as a molecular chaperone is exemplified in flying insects, including the fruit fly *Drosophila* and moth *Bombyx mori*, where CCS lacks the N-terminal ATX1 Cu-binding site [49, 50]. Insect CCS does not transfer Cu to SOD1, but promotes SOD1 protein stability [50], underscoring its role as a molecular chaperone.

Overall, nature has designed CCS dII across eukaryotes to bear sufficient resemblance to Cu/Zn SOD to facilitate CCS-SOD1 docking as well as promote an important molecular chaperone activity to CCS. CCS has also be reported to provide Cu to non-SOD1 targets such as the anti-apoptosis protein XIAP and the MEK1 signaling kinase [51, 52], and in these cases, CCS may purely function as Cu donor as opposed to molecular chaperone. With no Cu site in CCS dII, metal transfer from CCS to SOD1 occurs in just one direction. The importance of this one-way ticket for Cu is underscored in very recent studies of a R163W mutant variant of human CCS linked to a rare neurodegenerative disorder. R163W disrupts Zn binding in CCS dII such that Cu(I) can now bind to dII and pull Cu out of SOD1, creating an “anti-chaperone” effect [53].

It is believed that SOD1 and CCS diverged from a common ancestor early in eukaryotic evolution since nearly all eukaryotic organisms contain both molecules [47]. By comparison, two other classes of SOD1-like molecules appeared ≈1 billion years ago in the eukaryote supergroup known as opisthokonts, comprising animals and fungi [54]. These opisthokont specific proteins include the Cu-only SODs of the fungal kingdom and Cu-only SOD repeat proteins (CSRP) of animals.

## Cu-only SODs

The family of fungal Cu-only SODs contains all the hallmarks of Cu/Zn SOD enzymes (beta-barrel fold, intact Cu site, and intramolecular disulfide) with two noteworthy exceptions: fungal Cu-only SODs lack both a functional Zn binding site and extended loop VII/ESL [55–57]. As a result of an abbreviated ESL, the active site Cu ion is surface exposed [55–57] (Fig. 1C). Mycobacteria have also evolved with a Cu-only SOD; however this SOD retains the full length loop VII/ESL and the Cu containing active site is closed [58]. The fungal Cu-only SODs and the related CSRP family of SOD-like proteins in animals (see ahead) are uniquely designed with an open access Cu site.

Without the extended ESL to guide superoxide to the catalytic Cu, one might predict poor enzymatic activity for Cu-only SODs. But on the contrary, fungal Cu-only SODs are capable of disproportionating superoxide at rates that approach diffusion limits, just like their Cu/Zn SOD cousins [55–57]. Although unable to bind Zn, certain attributes of the Cu/Zn SOD Zn site are retained in Cu-only SODs. First, a Zn binding aspartate of Cu/Zn SODs is conserved in Cu-only SODs and is repurposed to orient a Cu-binding histidine through a water molecule (Fig. 1C). A second would-be Zn ligand is a glutamate in Cu-only SODs, which through hydrogen bonding, helps orient another Cu-binding histidine, the so-called “bridging histidine” of Cu/Zn SODs connecting Cu and Zn atoms (Fig. 1A, C). In Cu-only SODs, these aspartate and glutamate remnants of the Zn site promote pH independent catalysis and Cu-binding retention under acidic to slightly basic conditions [57]. For the purposes of this review, we shall refer to this signature pair of residues as “E110/D113” based on corresponding positions in Cu-only SOD5 of *C. albicans* [57] (Fig. 1C).

The ≈25 kDa Cu-only SODs with open active sites are only found in the fungal kingdom and related oomycete species, and in all cases, the SODs are extracellular, tethered to the cell wall or plasma membrane by GPI anchors or secreted into the extracellular space [57]. Fungi also express the bimetallic Cu/Zn SOD, but these are exclusively intracellular in the fungal kingdom, sharply contrasting animals, plants, and protozoans where Cu/Zn SODs can either be intracellular or extracellular [59–61]. Why are Cu-only SODs exclusively outside the cell? Clues may be obtained from examining the catalytic Cu-site. Cu-only SODs bind Cu with substantially lower affinity than Cu/Zn SODs [62, 63] and with the poor availability of Cu inside eukaryotic cells, Cu-only SODs may have insufficient metal binding affinity to effectively compete for Cu. In fact, unlike extracellular Cu/Zn SODs of animals that are activated with Cu inside the cell and are secreted in a fully metallated form, Cu-only SODs do not acquire Cu from intracellular sources. These enzymes arrive at the fungal cell surface as apo-protein and are activated by extracellular sources of Cu [56, 63]. With no known Cu chaperones and a readily accessible metal site, one might expect abundant mis-metallation of Cu-only SODs with non-Cu metals. However, Cu-only SODs are remarkably selective for binding Cu, and neither Zn, Fe or Mn will bind the active site under physiological conditions [56, 63]. This is in sharp contrast to the bimetallic Cu/Zn SODs, where Zn can occupy the Cu site or vice versa, at least *in vitro* [64–67]. The Cu-only SOD may be the perfect extracellular SOD, in that its activity is not restricted by the availability of intracellular Cu, as with all other cuproenzymes.

## Cu-only SOD enzymes and mimetics in health and disease

A number of fungal species are highly pathogenic in humans, and other species represent threats to the agriculture industry. During infection of animals or plants, fungi use their Cu-only SODs to thwart the attack of the host immune system involving ROS. These fungal enzymes readily degrade O_2_^•−^ produced by animal macrophages and neutrophils [68–70] and protect the fungi from killing by these host immune cells [68–73]. Cu-only SODs are regulated in pathogenic fungi according to virulence [74] and Cu availability [75], and most importantly, have been shown *in vivo* to be essential for virulence of wide-spread fungi that infect mammals [62, 69, 72, 76], plants [77, 78], and insects [79]. Because of their driving role in fungal pathogenesis, Cu-only SODs are a promising new antifungal target [80]. What makes them particularly attractive for drug development is their extracellular location providing easy access for chemical inhibitors, and the strong divergence in Cu-only SOD structure from the host Cu/Zn SODs. Preliminary screens for chemical inhibitors have revealed a number of metal binding compounds that readily inhibit fungal Cu-only SODs, but not the animal host Cu/Zn SOD [62, 63]. With the rising numbers of emergent and drug-resistant fungal pathogens [81, 82], the development of new antifungals that target Cu-only SODs is timely and important.

The Cu-only SOD enzyme is not just an attractive anti-fungal target, but mimics of these enzymes may prove applicable in medicine. Pecoraro and colleagues have recently engineered mimics of Cu-only SODs using rationally designed peptide-based Cu-binding scaffolds [83, 84]. These molecules are indeed reactive with superoxide and have revealed new insight into active site mechanism of Cu-only SODs [83, 84]. Cu-only SOD mimetics have also been designed in the form of nanozymes, and these nanomaterial-based mimics are effective anti-inflammatory agents in animal models of sepsis [85]. Future studies can examine more broadly the potential application of small molecule mimics of Cu-only SOD in inflammatory-based disease states.

## The Cu-only SOD variation in animals: CSRP

As mentioned above, Cu-only SODs are believed to have appeared early in the evolution of the eukaryotic supergroup Opisthokont, forming the fungal and animal kingdoms [54]. Fungi retained the SOD as a single ≈25 kDa enzyme, while in animals, the primordial Cu-only SOD underwent gene amplification events resulting in large 100 + kDa polypeptides containing 4–12 tandem repeats of Cu-only SOD-like domains, separated by very short linkers [55, 86]. We named these proteins CSRP for Cu-only SOD repeat protein [55], also known in fish as CUSR or in amphibians as SOD4 [87]. As with Cu-only SODs, each CSRP domain retains the trademark Cu/Zn SOD beta barrel fold and intramolecular disulfide, but lacks the Zn site and extended ESL/loop VII. Many but not all retain the aforementioned E110/D113 signature of Cu-only SODs and an intact Cu-binding site. Just like Cu-only SODs of fungi, animal CSRPs are predicted to be extracellular proteins either attached to the cell surface by GPI anchors or secreted [55, 86, 87].

**Figure 2:**
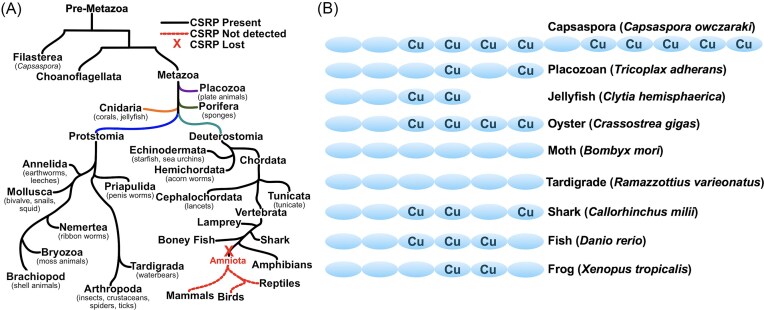
The evolution of CSRP across eukaryotes. (A) Simplified phylogenic tree of organisms with genomes that encode CSRP-like proteins. This tree includes pre-metazoan and metazoan species and not all phyla are displayed. Major metazoan phyla are color coded. The loss of CSRP genes in vertebrate amniotes is indicated by a red X. To date, no CSRP sequences have be identified in the available genomes for Protostomia Rotifera (wheel animals), Platyhelmintha (flatworms), and Nematoda (round worms). (B) Cartoon illustrating number of Cu-only SOD-like protein domains and the presence of intact Cu sites in CSRP of select organisms as determined by both primary sequence analyses and AlphaFold predictions [90–93]. All repeats possess the beta-barrel fold and conserved disulfide, although an alternative disulfide location has been identified in domain 6 of frog CSRP, but not CSRP of other amphibians, e.g. Axolotl (Table S1). The individual CSRP sequences can be retrieved through accession numbers provided in Table S1.

CSRP emerged at early stages in animal evolution, as the gene is present in *Capsaspora owczarzaki*, a single celled protist believed to be a unicellular ancestor to multicellular animals [55, 86, 87]. CSRP is found within numerous phyla of the animal kingdom, but not all species express CSRP [86] (Fig. 2A). Most notable, CSRPs are completely excluded from amniotes including birds, reptiles, and mammals, but prevalent among marine organisms and insects [55, 86, 87] (Fig. 2A). CSRP-producing organisms range from the primitive two-dimensional placozoans to marine invertebrates and insects, up to vertebrate jawless lampreys, and jawed sharks, fishes, and amphibians [55, 86–89] (Fig. 2A). A list of representative organisms reported with CSRPs is in Table S1.

**Figure 3:**
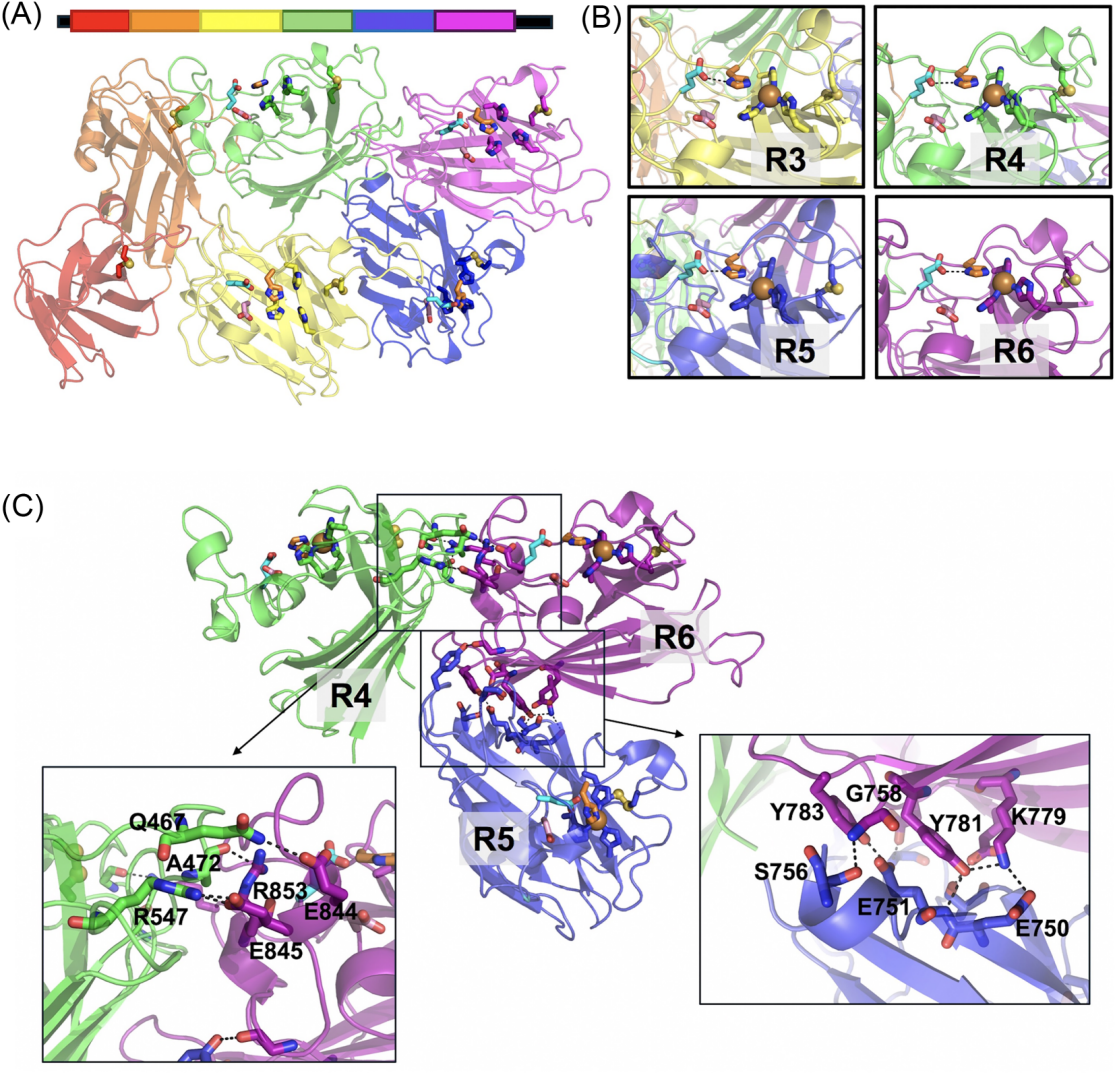
Structural predictions for CSRP. (A) Top: Cartoon showing organization of *Crassostrea gigas* CSRP. The N-terminal secretion signal and C-terminal GPI anchoring sites are shown as black lines and each predicted Cu-only SOD-like repeat is shown in a discrete color. Bottom: The 3D structure of apo *C. gigas* CSRP (A0A8W8N9N3) as predicted by AlphaFold [90–93], using the same color scheme depicted in Top. The N-terminal secretion signal and GPI-anchoring peptides are omitted for clarity. Based on the AlphaFold predicted local distance difference test (pLDDT) [104], the various *C. gigas* CSRP linkers score in either the very high (pLDDT > 90) or high (90 > pLDDT ≥ 70) range. (B) Prediction of Cu binding to *C. gigas* CSRP repeats 3–6 (R3-R6) as simulated by AlphaFold3 when four molar equivalents of Cu^2+^ are added per mole CSRP. (C) Protein-protein interactions between *C. gigas* CSRP R6 (purple), R4 (green), and R5 (blue) as modeled by AlphaFold. Inserts identity polar contacts at repeat interfaces. R6-R4 interactions involve a late region of the R6 extended Zn-loop and R4 residues in a loop spanning β-sheets 3 and 4 and directly preceding β-sheet 5. R6-R5 interactions involve R6 β-sheets 2 and 3 and R5 residues at the C-terminus of β-sheet 8 and connecting β-sheets 6 and 7.

CSRP was originally described in many organisms as having four-tandem Cu-only SOD-like repeats preceded by a long N-terminal region of unknown nature [55]. However, with the recent advent of AlphaFold, a more complete picture of CSRP has emerged that shows many CSRPs having six, not four repeats, with the previously unknown N-termini harboring a pair of Cu-only SOD backbone domains. The repeats are consistently separated by very short linkers, typically of five to nine residues in length. Fig. 3A illustrates CSRP domain analysis carried out by AlphaFold [90–93] using as example, CSRP from the oyster *Crassostrea gigas* [87, 94]. The model predicts six interacting Cu-only SOD-like domains, all containing the trademark eight stranded beta barrel fold and conserved disulfide, with the third, fourth, fifth, and sixth repeats each exhibiting predicted intact Cu sites and the E110/D113 signature of Cu-only SODs (Fig. 3A). To model Cu binding, various molar equivalents of Cu were simulated in the AlphaFold structure of *C. gigas* CSRP [93]. With a single Cu equivalent, the metal was predicted to bind the active site of the sixth repeat (R6), and with four molar equivalents, R3–R6 each contained single Cu atoms bound to the predicted Cu sites (Fig. 3B). No Cu-binding sequences outside of the four canonical sites where revealed, even when > 4 Cu atoms were modeled in the AlphaFold structure (Fig. S1A). These AlphaFold studies bolster primary sequence predictions on Cu-binding properties of CSRP, and can guide future biochemical and biophysical analysis of these cuproproteins.

The exact number of Cu-only SOD repeats and occurrence of intact Cu sites varies depending on the animal phyla examined. The most primitive CSRP of the unicellular Capsaspora is the largest identified thus far, containing 12 Cu-only SOD repeats, nine of which retain an intact Cu site (illustrated in Fig. 2B). CSRP then saw a decrease in repeats with evolution of multicellular animals, most containing six repeats with the notable exception of smaller CSRPs with four repeats in Cnidaria (jelly fish and coral). In all cases examined thus far, the N-terminal one or two repeats have lost the Cu-site (Fig. 2B).

Why has nature assembled the Cu-only SOD as tandem arrays on a single CSRP polypeptide? As suggested by Tan et al., repeated SOD domains may add functional versatility, allosteric regulation, and/or structural stability to CSRP [86]. Analogous to CCS dII (see above), the non-Cu-binding repeats in CSRP may act as molecular chaperones, helping to properly fold the CSRP polypeptide. Indeed one can see in the AlphaFold prediction of oyster CSRP, protein-protein interactions between multiple repeats, including the two non-Cu-binding domains at the N-terminus (Fig. 3A). In a detailed inspection of these protein-protein interactions, the contacts appear very different from the dimeric interface of the Cu/Zn SOD1 homodimer. SOD1 dimer formation is known to be driven largely by hydrophobic interactions involving β-sheets 1 and 8 and early segments of the Zn-loop (Fig. S1B) [95, 96]. In the model of *C. gigas* CRSP, R5 interactions with R4 and with R6 are driven by charged and aromatic residues, and involve regions of the beta barrel core distinct from that in SOD1 homodimers (Fig. 3C). Future studies are needed to determine the significance of these predicted protein-protein interactions in CSRP stability and function.

## The function of CSRP: tales from oysters, tardigrades, and frogs

Since we first described CSRP in 2018 [55], the function of this protein family has largely remained enigmatic. Are these SOD enzymes or do they operate in another capacity? Very recently, Tan and colleagues have succeeded in isolating recombinant CSRP from the oyster *Crassostrea sikamea* and demonstrated the protein indeed is capable of disproportionating superoxide anion [86]. At least in mollusks, CSRP is a SOD enzyme. Oyster CSRP is expressed throughout development [97] and is abundant in the mantel organ forming the exterior soft layer of the organism [86]. As the mantel is directly exposed to the aquatic environment, CSRP has been proposed to act as an antioxidant to help bivalves adapt to stressful and fluctuating habitats [86, 97]. It is important to note that in oysters, CSRP appears as the only extracellular active SOD. Oysters also produce an extracellular Cu/Zn SOD protein known as dominin, but dominin lacks an intact Cu site and is devoid of SOD activity [97, 98]. The SOD-like dominin functions in immune recognition of pathogenic bacteria [99], while CSRP handles extracellular superoxide [86].

Similar to oyster dominin, some CSRPs may operate independent of Cu binding and SOD activity. In arthropods including the *Bombyx mori* moth and arthropod-like tardigrades, the six domains of CSRP lack any obvious Cu site and are not predicted to have SOD activity (Fig. 2B). Nevertheless, these CSRPs are expressed [49, 89] and projected to fold in precisely the same manner as Cu-binding CSRPs. As seen in the overlay of tardigrade CSRP (no Cu-binding repeats) versus oyster CSRP (four Cu-binding repeats), the structures predicted by PyMOL are quite similar, with a root mean square deviation of the atomic positions of 2.1 (Fig. S1C) (PyMOL Molecular Graphics System, Version 3.0. Schrödinger, LLC). These Cu-less CSRPs may function similar to oyster dominin in immune responses or other extracellular capacities yet to be determined.

Lastly, clues as to CSRP function may be obtained by examining its specific distribution among the animal kingdom. The gene was lost during evolution of amniotes including mammals, birds, and reptiles (Fig. 2A). Interestingly, amniotes have lost the high capacity for tissue regeneration [100]. The ability to regenerate limbs and organ tissue is a common feature among anamniote vertebrates including amphibians and boney fish, and is also prevalent among various invertebrates. The inability of mammals to regenerate tissue is a major issue in the clinics, particular with cardiovascular disease, and much research is devoted towards understanding the keys to tissue regeneration in anamniotes.

In a recent attempt to identify anamniote factors responsible for tissue regeneration, Lyubetsky et al. conducted a large-scale search for genes absent in amniotes, yet retained in fish and amphibians and abundantly expressed in these anamniotes during wound healing. One such gene identified was CSRP [87]. The authors went on to test whether CSRP is important for tissue regeneration by knocking down CSRP expression in the frog *Xenopus laevis*. Indeed CSRP was found to be essential for tissue regeneration in a tadpole tail amputation model [87]. Thus, at least in *Xenopus*, CSRP promotes wound healing. In vertebrate fish and amphibians, wound healing is associated with bursts of ROS production [101–103], and an extracellular CSRP with SOD activity could signal tissue regeneration through ROS. It is also possible that CSRP promotes wound healing through a mechanism that does not involve ROS since not all CSRP molecules are predicted to have SOD activity.

## Concluding remarks

Since first discovered by Irwin Fridovich, the Cu/Zn SOD has taken on many new identities in eukaryotic cells. Early in eukaryotic evolution, a primordial Cu/Zn SOD lost its active site and was fused to Cu-binding sequences creating the CCS Cu chaperone that can also function as a molecular chaperone. In the Opisthokonta supergroup that generated fungi and animals, a Cu/Zn SOD molecule lost its Zn binding capacity and ESL/loop VII, exposing the Cu site and forming Cu-only SODs of the fungal kingdom, and tandemly amplified Cu-only SODs in animal CSRP. These changes in the SOD active site came with a price of a lowered Cu-binding affinity, but Cu-only SODs and CSRPs adapted by becoming exclusively extracellular, so they need not compete for scant intracellular Cu. Much new light has recently been shed on the function on these proteins. In pathogenic fungi, Cu-only SODs thwart off host attacks of ROS and promote pathogen virulence, and in bivalves including oysters, CSRP is believed to provide antioxidant protection in hostile environments. Most exciting was the recent finding of amphibian CSRP functioning in tissue regeneration. The upcoming years are expected to see many new advances in our understanding of Cu-only SODs and CSRP. Studies focused on the development of inhibitors for Cu-only SODs represent promising new avenues to treat and prevent deleterious fungal infections in humans and the agricultural industry. Very little is currently understood regarding the biochemical, biophysical and cell biology properties of CRSP. For example, do the individual CSRP repeats indeed bind Cu as predicted from bioinformatic studies, and how do CSRP molecules obtain Cu? Based on their predicted extracellular location and surface-exposed Cu sites, CSRP molecules are expected to obtain Cu in the same fashion as Cu-only SODs, i.e. arriving at the cell surface in an apo form followed by activation through extracellular sources of Cu. Future studies are needed to understand the metal binding properties of the CSRP family of proteins. Additionally, research into understanding the role of CSRP in tissue and limb regeneration is particularly worthy of investigation. Although CSRP clearly has SOD activity in some organisms, this is not the entire story, as several organisms express CSRP variants that have no obvious Cu-binding sites. There is much to be learned in the years to come on these curious derivatives of Cu/Zn SOD.

## Supplementary Material

mfag007_Supplemental_Files

## Data Availability

The data underlying this article are available in the article and in its online supplementary material.
