# Supplementary material for: From hemocuprein to CSRP: the many faces of Cu/Zn superoxide dismutase
Source: Metallomics. 2026 Feb 18;18(1):mfag007. doi: 10.1093/mtomcs/mfag007 (PMC13017621; doi:10.1093/mtomcs/mfag007)
Supplement: mfag007_Supplemental_Files [file mfag007_supplemental_files.zip › Suppl_Data Fig S1.pdf]

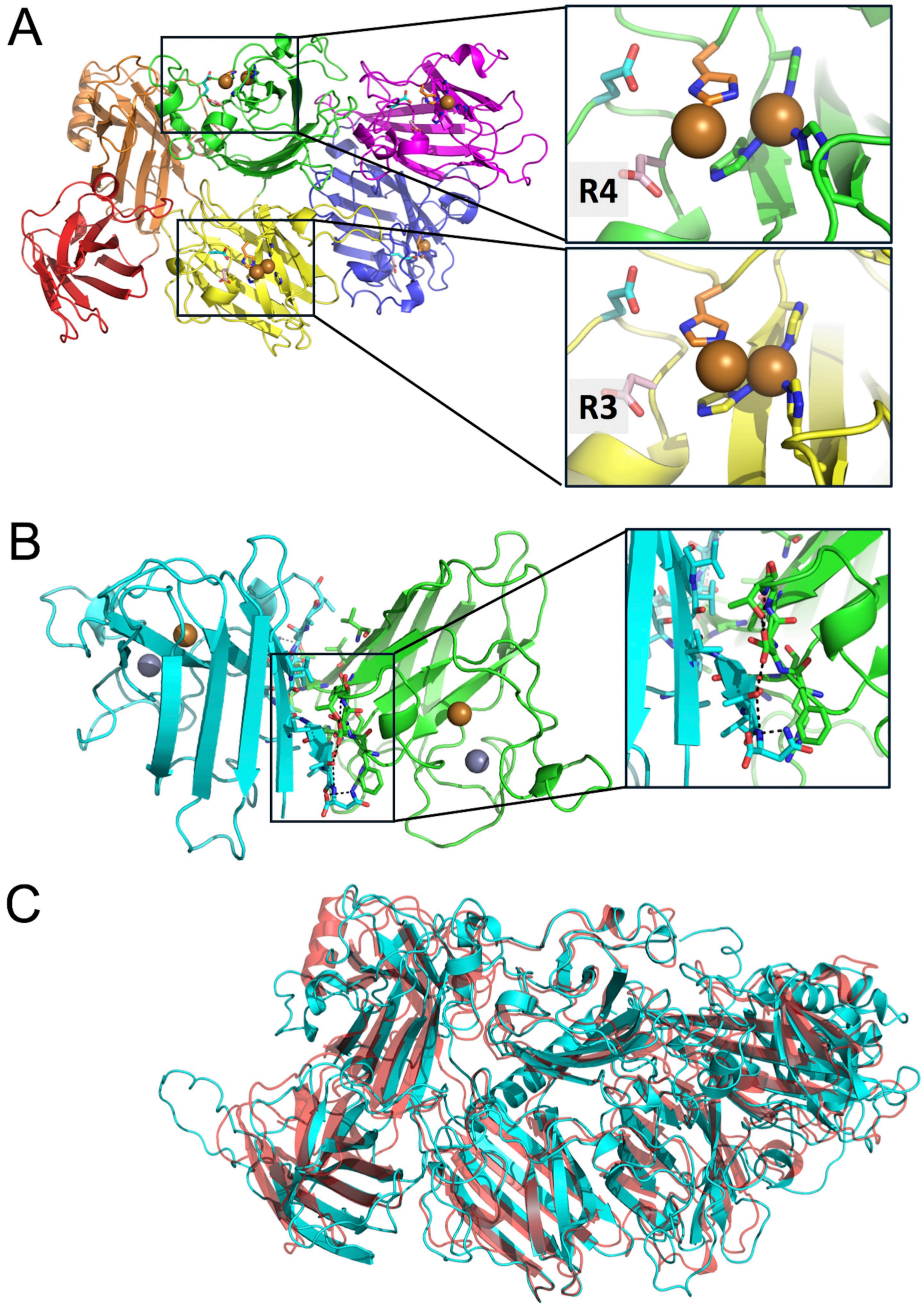

Supplementary Data Figure S1: AlphaFold modeling of CSRP and the structure of Cu/Zn SOD1 (A) Cu binding to *C. gigas* CSRP as predicted by AlphaFold3 using 6 Cu equivalents per mole CSRP. In the simulation, all 6 Cu ions bind the four canonical metal sites in R3-R6, with putative di-Cu sites in R3 and R4. Di-Cu sites have not been reported for any Cu-SOD proteins, and their possible existence in CSRP warrants biophysical analysis. (B) Structure of *Saccharomyces cerevisiae* Cu/Zn SOD (PDB:2JCW) [1], with dimer interface expanded in the inset. Residues within an early Zn loop region of the green SOD1 monomer interact with residues in β-sheets 1 and 8 of the cyan SOD1 monomer. Isoleucine residues on the cyan monomer β-sheets and the threonine sidechain of the green monomer result in a hydrophobic dimer interface. (C) Overlay structural comparison of the AlphaFold predicted 3D structures of CSRP from *C. gigas* (red) and the tardigrade *Ramazzottius varieonatus* (cyan) (A0A1D1VWV6). Based on PyMOL alignments of the core 6 repeat units, the root mean square deviation of the atomic positions (RMSD) for CSRP from *R. varieonatus* (residues 43-1011) and *C. gigas* (residues 23-915) is 2.1 over 681 C-alpha atoms.

1. Hart PJ, Balbirnie MM, Ogihara NL, Nersissian AM, Weiss MS, Valentine JS, Eisenberg D. A structure-based mechanism for copper-zinc superoxide dismutase. *Biochemistry* 1999;**38**(7):2167-78. doi: 10.1021/bi982284u bi982284u [pii]
